# 

**Published:** 1893-07-01

**Authors:** 


					HOSPITAL. ' July 1st, ;
PRICE Ttt/nBCNrc '  -Z WW ^Ss^, f WITH extra nursing supplement
No 353 V^l Y l\/ I I i X lonn I==l /H^ Per Annum. 8s. ?d., post free.
t^eBistered't ^ July 1st, 1893. JOl MontMy Parts, 6d.; post free, 94.
[Begin VI1 '?? v i u
m> "?= ?=S^> Ditto per Annmn, 8s., post free*
No, 353. Vol. XIV. Satueday,
July 1st, 1893.
[Twopence.

				

